# The Role of Cancer in the Risk of Cardiovascular and All-Cause Mortality: A Nationwide Prospective Cohort Study

**DOI:** 10.3389/ijph.2023.1606088

**Published:** 2023-10-19

**Authors:** Ruihuan Shen, Jia Wang, Rui Wang, Yuqing Tian, Peiyao Guo, Shuhui Shen, Donghao Liu, Tong Zou

**Affiliations:** ^1^ Department of Cardiology, Beijing Hospital, National Center of Gerontology, Institute of Geriatric Medicine, Chinese Academy of Medical Sciences and Peking Union Medical College, Beijing, China; ^2^ Graduate School of Peking Union Medical College, Beijing, China; ^3^ Department of Cardiology, Beijing Hospital, National Center of Gerontology, Institute of Geriatric Medicine, Peking University Fifth School of Clinical Medicine, Beijing, China

**Keywords:** all-cause, cardiovascular, mortality, cancer, NHANES

## Abstract

**Objectives:** Evidence on cardiovascular-related and all-cause mortality risks in a wide range of cancer survivors is scarce but needed to inform prevention and management.

**Methods:** We performed a nationwide prospective cohort study using information from the Continuous National Health and Nutrition Examination Survey (NHANES) in the United States and the linked mortality follow-up files, available for public access. A propensity score-matched analysis with a 1:1 ratio was conducted to reduce the baseline differences between participants with and without cancer. The relationship between cancer status and the cardiovascular-related and all-cause mortality risk was examined using weighted Cox proportional hazards regression. Independent stratification analysis and cancer-specific analyses were also performed.

**Results:** The study sample included 44,342 participants, aged 20–85, interviewed between 1999 and 2018. Of these, 4,149 participants had cancer. All-cause death occurred in 6,655 participants, of whom 2,053 died from cardiovascular causes. Propensity-score matching identified 4,149 matched pairs of patients. A fully adjusted Cox proportional hazards regression showed that cancer was linked to an elevated risk of cardiovascular-related and all-cause mortality both before and after propensity score matching. Stratification analysis and cancer-specific analyses confirmed robustness of results.

**Conclusion:** Our study confirmed that cancer was strongly linked to cardiovascular-related and all-cause mortality, even after adjusting for other factors that could impact a risk, including the American Heart Association (AHA)’s Life’s Simple 7 cardiovascular health score, age, sex, ethnicity, marital status, income, and education level.

## Introduction

Cardiovascular disease (CVD) exhibits a significant prevalence and stands as the leading cause of death in certain cancer patients [1]. CVD and cancer possess a multitude of shared risk factors, including but not limited to diabetes, hypertension, obesity, hyperlipidemia, and lifestyle factors [2, 3]. Moreover, these two conditions share pathophysiologic mechanisms that potentially render patients susceptible to both conditions. Furthermore, certain cancer treatments may induce cardiac toxicity, thereby augmenting the likelihood of CVD and CVD-related mortality in cancer patients [4].

In recent decades, significant progress has been achieved in the field of cancer screening, diagnosis, and therapeutic interventions, leading to a notable increase in the survival rates of numerous cancer patients beyond the 5 years mark [5–8]. Consequently, there is an anticipated rise in the population of cancer survivors. Nonetheless, individuals diagnosed with cancer frequently experience a substantial burden of chronic health conditions resulting from the long-term effects of the disease and its treatments. Moreover, their extended lifespan exposes them to a higher risk of non-cancer-related mortality surpassing that of cancer-related mortality [9, 10].

Despite the growing acknowledgment of a strong association between CVD incidence and cancer, there is a dearth of prospective studies that have thoroughly evaluated the elevated risk of CVD mortality among individuals diagnosed with cancer. Therefore, the objective of this population-based multicycle cross-sectional study intended to assessed the excess risk of CVD mortality among participants with cancer, with the aim of providing insights for the enhancement of prevention and management strategies.

## Methods

### Database

The National Center for Health Statistics (NCHS) of the Centers for Disease Control and Prevention (CDC) launched several cycles of the United States cross-sectional Continuous NHANES between 1999 and 2018 [11].

The NHANES utilized a complex, stratified, multistage probability cluster design to create a nationally-representative survey of the health and nutritional status of the non-institutionalized civilian population in the United States, with detailed information on the survey methods and analytic guidelines made publicly available [12]. The nutritional and health condition data were acquired through a series of home interviews, examinations, and laboratory measurements. The NHANES interview includes demographic, socioeconomic, dietary, and health-related questions. The examination component included medical, dental, and physiological assessments, and laboratory tests were administered by highly-trained medical personnel.

Moreover, the NCHS has connected many demographic surveys to death certificate information from the National Death Index (NDI). The files were processed to minimize the likelihood of participant identification, and the public-use versions linking mortality follow-up data for adult participants from the date of survey participation through 31 December 2019 were made available [13].

### Study Design and Population

The Continuous NHANES data were collected from 1999 to 2018 in 2 years increments for the initial sample. The medical conditions section (prefix MCQ) provides self-reported personal interview data on a broad range of health conditions for children and adults. Only participants aged 20–85 with available demographic data who answered the following self-reported questions were included: “Ever told you had cancer or malignancy?” (Question MCQ220) and “What kind of cancer?” (Questions MCQ230A-D). Responses marked “missing,” “refused,” or “do not know” were regarded as missing in the original NHANES surveys. Especially, male breast cancer was excluded in our study. Participants lacking information for follow-up and any of the study covariates specified below were excluded from the data analysis.

### Data Collection and Weight Selection

Demographic, physical measures, and comorbidities data were recorded. Demographic data, such as age, sex, ethnicity, marital status, smoking status, and educational level, and information on certain comorbid conditions [e.g., congestive heart failure (CHF), coronary heart disease (CHD), angina, heart attack (also called myocardial infarction, MI), stroke, hypertension, diabetes mellitus (DM), and hyperlipidemia] were obtained during the home interviews. Trained health technicians and interviewers delivered standardized body measurements [e.g., body mass index (BMI)] to survey participants at the mobile examination facility (MEC). The NHANES 1999–2018 MEC examination data weights were used in all analyses to account for stratification and clustering because of the complex sample design.

### Independent Variable

#### Ascertainment of Cancer and Its Type

Participants who responded “yes” to the question “Have you ever been told by a doctor or other health professional that you had cancer or a malignancy of any kind?” during the home interview were deemed as having cancer or malignancy.

The cancer type was defined by the code or value the participants entered under the question “What kind of cancer?”

### Follow‐Up and Outcomes

Follow-up lasted from the interview date to the last follow‐up, 31 December 2019, or the date of death, whichever came first. Records from the NDI provided information on the causes of death for the included participants. The mortality outcomes were defined according to the International Statistical Classification of Diseases and Related Health Problems, 10th Revision (ICD-10) codes recorded as the leading cause of death.

The study endpoints were all-cause, and cardiovascular-related death (I00–I09, I11, I13, I20–I51, and I60–I69). Cardiovascular-related death encompassed diseases of heart (I00–I09, I11, I13, I20–I51) and cerebrovascular diseases death (I60–I69).

### Covariates

These covariates were deemed necessary to account for differences in cardiovascular-related and all-cause mortality between participants with and without cancer. The analysis included age and the Life’s Simple 7 cardiovascular health score as continuous variables. The Life’s Simple 7 criteria, devised by the AHA to describe ideal cardiovascular health, include not smoking, regular physical activity, healthy diet, maintaining normal body weight, and controlling cholesterol, blood pressure, and blood glucose levels. The Life’s Simple 7 cardiovascular health score can vary from 0 to 14 (0 being the worst score and 14 the optimal one) and was calculated by adding the number of ideal health metrics achieved. Sex was dichotomized into male and female. Ethnicity was classified as White or Non-White. The marital status category included “Living with a Spouse or Partner” and “Living without a Spouse or Partner.” The educational background was specified as a college graduate or above, some college or AA degree, high school graduate, 9–11th grade, and under 9th grade. The income categories included low [poverty income ratio (PIR) < 1.3], middle (PIR, 1.3–3.5), and high (PIR ≥ 3.5) [14, 15]. The smoking status categories were former smoker, current smoker, and never smoked. BMI was classified as low (<18.5 kg/m^2^), normal (18.5–25.0 kg/m^2^), or overweight (≥25.0 kg/m^2^) [16].

### Comorbid Conditions

Information on comorbidities was self-reported. Participants were considered as having a comorbidity (CHF, CHD, angina/angina pectoris, heart attack, or stroke) when answering “yes” to the question “Have you ever been told by a doctor or health professional that you have …?”

Hypertension was diagnosed based on the following blood pressure/cholesterol questions: BPQ020: “Have you ever been told that you had high blood pressure?” BPQ030: “Have you been told that you had high blood pressure 2+ times?” BPQ040A: “Are you taking a prescription for hypertension?”; using anti-hypertension drug; judging hypertension on average blood pressure. Average blood pressure was calculated by the following protocol: 1. If only one blood pressure reading was obtained, that reading was used as the average. 2. If more than one blood pressure readings were available, the first reading was always excluded from the average. 3. If only two blood pressure readings were obtained, the second one was used as the average. 4. If all the diastolic readings were zero, the average was zero.

The diagnostic criteria for diabetes were as follows: a doctor told you that you have diabetes; a hemoglobin A1c of > 6.5%; a random blood glucose concentration of ≥11.1 mmol/L; a 2 h oral glucose tolerance test (OGTT) blood glucose concentration of ≥11.1 mmol/L; use diabetes medication or insulin. Hyperlipidemia was defined as an elevated triglyceride (≥150 mg/dL) or cholesterol (total cholesterol ≥200 mg/dL [5.18 mmol/L], low-density lipoprotein cholesterol ≥130 mg/dL [3.37 mmol/L], or high density lipoprotein cholesterol <40 mg/dL [1.04 mmol/L] in males or <50 mg/dL [1.30 mmol/L] in females) level or the use of cholesterol-lowering agents [17].

### Statistical Analysis

Categorical variables are expressed as weighted proportions and corresponding 95% confidence intervals (CIs). Design-based χ^2^ tests investigated the association between categorical variables and cancer or CVD status.

The Kolmogorov-Smirnov test of normality assessed the distribution (normal or non-normal) of continuous variables. Normally-distributed continuous variables are presented as weighted means and associated standard errors (SEs), while non-normally distributed variables are presented as weighted medians and associated interquartile ranges (IQRs). The two-sample Student’s t-test compared normally distributed variables, while the Mann-Whitney U test compared non-normally distributed variables.

Three weighted Cox proportional hazards regression models were used to estimate the associations of cancer and cancer types with the all-cause and cardiovascular-related mortality probabilities after controlling for possible confounding factors. Accumulating evidence suggests disparities in risk factors of CVD in the general population, depending on age, sex, ethnicity, marital status, educational level, income, and the AHA’s Life’s Simple 7 cardiovascular health score [18–29]. Therefore, the multivariable model included the following confounders based on previously published studies: age (<50 or ≥50 years), sex (male or female), ethnicity (White or Non-White), marital status (living with a spouse/partner or living without a spouse/partner), PIR [low (<1.3), middle (1.3–3.5), or high (≥3.5) income], educational level (under 9th grade, 9–11th grade, high school graduate, some college or AA degree, or college graduate or above), and the AHA’s Life’s Simple 7 cardiovascular health score (continuous). The second and third adjusted models included robust adjustments for covariates thought to be potential confounders of the associations between cancer and all-cause or cardiovascular-related mortality. We performed similar exploratory analyses for the associations of the specific cancers with all-cause and cardiovascular-related mortality. The associations between cancer and the outcomes are presented as crude and adjusted hazard ratios (HRs) and their 95% confidence intervals (CIs).

Model 1 was an unadjusted model; Model 2 was adjusted for age, sex, ethnicity, marital status, PIR, and educational level. Model 3 further adjusted for the AHA’s Life’s Simple 7 cardiovascular health score.

Independent stratification analysis was performed to determine if the association between cancer and all-cause and cardiovascular-related mortality varied across subgroups in each covariate category. For analyses stratified by sex, we created a non-sex-related cancer category that included all cancers that could occur in both sexes, while excluding breast, ovarian, cervical, uterine, and prostate cancers. The Wald test calculated the *p* values for the interactions.

In order to mitigate the influence of initial disparities in demographic and comorbid conditions on the cardiovascular and all-cause mortality, we employed the 1:1 propensity score matching (PSM) with sampling weights technique to align the individuals in the cancer and non-cancer cohorts. The PSM approach validates the existence of preexisting dissimilarities through standardized differences. The fundamental concept underlying the propensity score is to substitute multiple covariates with a single score to equalize the distribution of covariates among participants with and without cancer. In order to mitigate selection bias, non-randomized studies employed a method akin to randomization by ensuring the equitable distribution of confounding factors. These factors encompassed age, sex, ethnicity, marital status, PIR, educational level, smoking status, BMI, hypertension, CVD, DM, hyperlipidemia, and LS7 score. The ratio value was set at 1, while the caliper value was established at 0.02. The LOVE plot (Absolute standardized differences plot) was a simple and straightforward way to summarize balance visually. Through the LOVE plot, we can see the changes and distribution of standardized mean differences (SMD) before and after matching.

We used R (Version 4.1.2; [30]) for statistical analysis. The complexity of the sampling design was considered in each analysis by specifying primary sampling units (PSUs), strata, and weights using the R package “survey” (Version 4.1-1). We used the MEC examination weights for all sample estimations [31–33]. Two‐sided *p* values < 0.05 were considered statistically significant.

## Results

### Sample Characteristics

From 1999 to 2018, the total number of people who participated in the NHANES program was 55,081. After excluding participants with missing demographic (*n* = 5,859), covariate data (*n* = 4,820) and follow-up information (*n* = 60), the study included 44,342 participants for the final statistical analysis, representing 191.04 million non-institutionalized United States residents with an average age of 46.90 ± 0.19 years. Of these, 22,362 (weighted proportion, 51.23%; 95% CI, 49.31%–53.14%) were female.

A total of 4,149 participants (weighted proportion, 9.50%; 95% CI, 8.94%–10.06%) had cancer, corresponding to 18.15 million adults in the general population. These included 639 with breast cancer (weighted proportion, 2.88%; 95% CI, 2.59%–3.17%), 111 with ovarian cancer (weighted proportion, 0.46%; 95% CI, 0.35%–0.56%), 282 with cervical cancer (weighted proportion, 1.51%; 95% CI, 1.27%–1.74%), 198 with uterine cancer (weighted proportion, 0.81%; 95% CI, 0.66%–0.96%), 686 with prostate cancer (weighted proportion, 2.01%; 95% CI, 1.79%–2.24%), 102 with lymphoma or Hodgkin’s disease (weighted proportion, 0.25%; 95% CI, 0.18%–0.31%), 279 with melanoma (weighted proportion, 0.80%; 95% CI, 0.67%–0.93%), 693 with non-melanoma skin cancer (weighted proportion, 2.09%; 95% CI, 1.87%–2.31%), 362 with unknown skin cancer (weighted proportion, 0.95%; 95% CI, 0.81%–1.08%), 93 with thyroid cancer (weighted proportion, 0.23%; 95% CI, 0.17%–0.30%), 122 with lung cancer (weighted proportion, 0.23%; 95% CI, 0.18%–0.28%), 311 with colon cancer (weighted proportion, 0.51%; 95% CI, 0.43%–0.60%), 89 with kidney cancer (weighted proportion, 0.15%; 95% CI, 0.11%–0.19%), and 115 with bladder cancer (weighted proportion, 0.19%; 95% CI, 0.15%–0.24%). These study participants correspond, respectively, to 2.82 million, 0.45 million, 1.47 million, 0.79 million, 1.88 million, 0.47 million, 1.53 million, 3.99 million, 1.81 million, 0.45 million, 0.44 million, 0.98 million, 0.29 million, and 0.37 million adults in the general population.

Over a median follow‐up of 109 months, all‐cause death occurred in 6,655 participants (weighted proportion, 10.73%; 95% CI, 10.06%–11.40%) and cardiovascular-related death occurred in 2,053 (weighted proportion, 3.14%; 95% CI, 2.86%–3.41%), corresponding to 20.50 and 6.00 million adults in the general population, respectively.

Table 1 presents the sociodemographic and clinical characteristics of the weighted population grouped by cancer. Compared to participants without cancer, those with cancer were more likely to be older, White, female, and with a lower Life’s Simple 7 cardiovascular health score and higher income and educational background, while participants without cancer were more likely to have never smoked and be living with a spouse or partner. Compared to the no-cancer group, participants in the cancer group had a greater risk of the following comorbidities: hypertension, CVD, CHF, CHD, angina, MI, stroke, diabetes, and hyperlipidemia.

**TABLE 1:** Baseline characteristics of study participants according to cancer status, continuous national health and nutrition examination survey, 1999 to 2018 before propensity score matching^a^ (The United States, 1999–2018).

Characteristic	Participants			*p*-value
	Total (*n* = 44,342)	No cancer (*n* = 40,193)	Cancer (*n* = 4,149)	
Age	46.90 (0.19)	45.29 (0.18)	62.17 (0.31)	<0.0001
Life’s Simple 7	8.01 (0.03)	8.08 (0.03)	7.33 (0.05)	<0.0001
Sex				<0.0001
Female	51.23 (49.31, 53.14)	50.62 (50.14, 51.10)	57.05 (55.33, 58.77)	
Male	48.77 (46.98, 50.56)	49.38 (48.90, 49.86)	42.95 (41.23, 44.67)	
Ethnicity (%)				<0.0001
White	69.27 (65.05, 73.50)	67.41 (65.34, 69.48)	87.01 (85.52, 88.50)	
Non-white	30.73 (29.37, 32.09)	32.59 (30.52, 34.66)	12.99 (11.50, 14.48)	
Marital Status (%)				0.004
Living with a Spouse or Partner	63.90 (61.02, 66.77)	63.62 (62.63, 64.62)	66.50 (64.55, 68.45)	
Living without a Spouse or Partner	36.10 (34.88, 37.33)	36.38 (35.38, 37.37)	33.50 (31.55, 35.45)	
Educational Level (%)				<0.0001
College Graduate or above	28.27 (26.50, 30.05)	27.85 (26.38, 29.33)	32.28 (29.81, 34.75)	
Some College or AA Degree	31.11 (29.79, 32.43)	31.10 (30.34, 31.86)	31.22 (29.25, 33.20)	
High School Graduate	24.01 (22.66, 25.36)	24.18 (23.33, 25.03)	22.37 (20.52, 24.23)	
9–11th Grade	11.03 (10.29, 11.76)	11.22 (10.56, 11.88)	9.23 (8.08, 10.37)	
Less than 9th Grade	5.58 (5.17, 5.99)	5.65 (5.22, 6.07)	4.90 (4.21, 5.59)	
Poverty Income Ratio (%)^b^				<0.0001
High Income	42.89 (40.61, 45.17)	42.37 (40.85, 43.89)	47.90 (45.37, 50.44)	
Middle Income	35.84 (34.19, 37.50)	35.80 (34.83, 36.77)	36.24 (34.21, 38.26)	
Low Income	21.26 (20.09, 22.44)	21.83 (20.76, 22.90)	15.86 (14.20, 17.52)	
Smoking Status (%)^c^				<0.0001
Never	53.67 (51.78, 55.56)	54.65 (53.62, 55.68)	44.27 (42.36, 46.18)	
Former	24.69 (23.37, 26.02)	23.20 (22.49, 23.91)	38.96 (36.98, 40.94)	
Current	21.64 (20.51, 22.76)	22.15 (21.35, 22.95)	16.77 (15.18, 18.35)	
Body Mass Index (%)^d^				0.26
Normal	29.59 (28.31, 30.86)	29.73 (28.91, 30.54)	28.25 (26.62, 29.88)	
Overweight	68.78 (66.12, 71.44)	68.65 (67.78, 69.52)	69.99 (68.36, 71.63)	
Low	1.64 (1.47, 1.80)	1.63 (1.46, 1.79)	1.76 (1.24, 2.27)	
CVD (%)	8.43 (7.90, 8.97)	7.29 (6.91, 7.67)	19.33 (17.78, 20.87)	<0.0001
CHF (%)	2.21 (2.02, 2.41)	1.83 (1.68, 1.98)	5.86 (5.05, 6.68)	<0.0001
CHD (%)	3.34 (3.04, 3.64)	2.87 (2.63, 3.11)	7.82 (6.75, 8.90)	<0.0001
Angina (%)	2.40 (2.15, 2.65)	2.09 (1.89, 2.29)	5.31 (4.38, 6.25)	<0.0001
Myocardial Infarction (%)	3.31 (3.02, 3.59)	2.85 (2.62, 3.08)	7.65 (6.75, 8.55)	<0.0001
Stroke (%)	2.76 (2.54, 2.97)	2.40 (2.22, 2.58)	6.17 (5.34, 7.01)	<0.0001
Hypertension (%)	36.85 (35.23, 38.46)	34.65 (33.82, 35.47)	57.82 (55.91, 59.73)	
Diabetes Mellitus (%)				<0.0001
No	80.75 (77.75, 83.75)	81.87 (81.23, 82.50)	70.11 (68.47, 71.75)	
Diabetes	12.60 (11.97, 13.22)	11.71 (11.26, 12.15)	21.09 (19.58, 22.59)	
IFG	4.25 (3.88, 4.62)	4.10 (3.76, 4.44)	5.68 (4.74, 6.62)	
IGT	2.40 (2.19, 2.61)	2.32 (2.13, 2.52)	3.12 (2.51, 3.73)	
Hyperlipidemia (%)	68.51 (65.65, 71.38)	67.50 (66.63, 68.36)	78.20 (76.33, 80.07)	<0.0001

Abbreviations: CHF, congestive heart failure; CHD, coronary heart disease; CVD, cardiovascular disease; IFG, impaired fasting glucose; IGT, impaired glucose tolerance.

^a^ Two-sided *p* values show results of univariate comparisons between participants with cancer and participants without cancer. The two-samples Student’s t*-*test was used for normally distributed variables, while Mann-Whitney U test was used for non-parametric variables. Design-based χ^2^ tests were employed to assess the associations of categorical variables with cancer status. Continuous variables with normality were presented as weighted means with associated standard errors, and variables without normality were presented as weighted median with associated interquartile range. Categorical variables were expressed as weighted proportions and corresponding 95% confidence intervals.

^b^ Categorized into the following three levels based on the poverty income ratio: low income (<1.3), middle income (1.3–3.5), and high income (≥3.5).

^c^ Categorized into the following three levels: never, smoked less than 100 cigarettes in life; former, smoked more than 100 cigarettes in life and smoke not at all; current, smoked more than 100 cigarettes in life and smoke some days or every day.

^d^ Divided into three categories: low (<18.5 kg/m^2^), normal (18.5–25 kg/m^2^), overweight (≥25 kg/m^2^).

**FIGURE 1:**
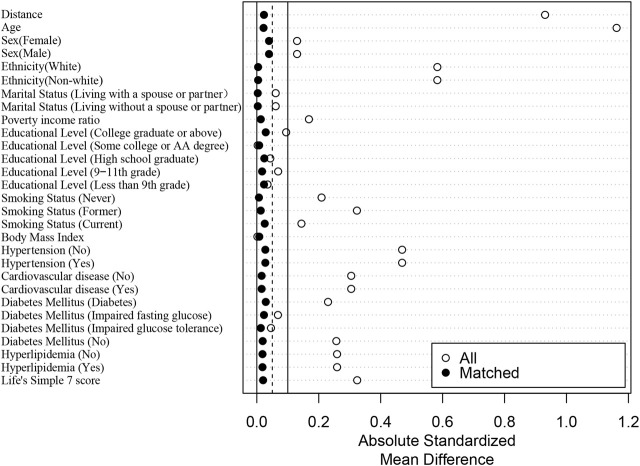
Absolute standardized differences plot (LOVE plot) (The United States, 1999–2018).

A 1:1 PSM analysis was conducted to mitigate bias between participants with and without cancer. Ultimately, a total of 8,298 participants were evaluated, with each subgroup consisting of 4,149 participants. The *p* values for all covariates exceeded 0.05, indicating a significant overlap in propensity scores between the two groups (Table 2). As can be seen from the LOVE plot in Figure 1, the balance is very poor before matching, and most of the variables are distributed in areas other than 0.1. After matching, the balance improves a lot, and the SMD distribution of all variables is within 0.1. Moreover, other details that show the improvements due to PSM were presented in Supplementary Tables S1–S3.

**TABLE 2:** Baseline characteristics of study participants according to cancer status, continuous national health and nutrition examination survey, 1999 to 2018 after propensity score matching^a^ (The United States, 1999–2018).

Characteristic	Participants		*p*-value
	No cancer (*n* = 4,149)	Cancer (*n* = 4,149)	
Age	61.86 (0.29)	62.17 (0.31)	0.44
Life’s Simple 7	7.37 (0.05)	7.33 (0.05)	0.46
Sex			0.18
Female	59.00 (57.08, 60.92)	57.05 (55.33, 58.77)	
Male	41.00 (39.08, 42.92)	42.95 (41.23, 44.67)	
Ethnicity (%)			0.85
White	86.87 (85.39, 88.36)	87.01 (85.52, 88.50)	
Non-white	13.13 (11.64, 14.61)	12.99 (11.50, 14.48)	
Marital Status (%)			0.91
Living with a Spouse or Partner	66.36 (64.31, 68.41)	66.50 (64.55, 68.45)	
Living without a Spouse or Partner	33.64 (31.59,35.69)	33.50 (31.55,35.45)	
Educational Level (%)			0.53
College Graduate or above	30.94 (28.49, 33.38)	32.28 (29.81, 34.75)	
Some College or AA Degree	31.57 (29.60, 33.55)	31.22 (29.25, 33.20)	
High School Graduate	23.37 (21.64, 25.11)	22.37 (20.52, 24.23)	
9–11th Grade	9.72 (8.34, 11.09)	9.23 (8.08, 10.37)	
Less than 9th Grade	4.40 (3.73, 5.06)	4.90 (4.21, 5.59)	
Poverty Income Ratio (%)^b^			0.76
High Income	47.08 (44.41, 49.74)	47.90 (45.37, 50.44)	
Middle Income	37.12 (35.06, 39.18)	36.24 (34.21, 38.26)	
Low Income	15.81 (14.07, 17.55)	15.86 (14.20, 17.52)	
Smoking Status (%)^c^			0.67
Never	43.93 (41.92, 45.94)	44.27 (42.36, 46.18)	
Former	38.35 (36.43, 40.27)	38.96 (36.98, 40.94)	
Current	17.72 (16.02, 19.42)	16.77 (15.18, 18.35)	
Body Mass Index (%)^d^			0.07
Normal	27.71 (25.88, 29.53)	28.25 (26.62, 29.88)	
Overweight	71.28 (69.41, 73.15)	69.99 (68.36, 71.63)	
Low	1.01 (0.62, 1.41)	1.76 (1.24, 2.27)	
CVD (%)	19.94 (18.49, 21.39)	19.33 (17.78, 20.87)	0.56
CHF (%)	5.26 (4.51, 6.01)	5.86 (5.05, 6.68)	0.27
CHD (%)	8.43 (7.29, 9.56)	7.82 (6.75, 8.90)	0.45
Angina (%)	6.00 (4.94, 7.05)	5.31 (4.38, 6.25)	0.27
Myocardial Infarction (%)	7.99 (7.04, 8.95)	7.65 (6.75, 8.55)	0.60
Stroke (%)	6.14 (5.25, 7.03)	6.17 (5.34, 7.01)	0.95
Hypertension (%)	56.46 (54.43, 58.49)	57.82 (55.91, 59.73)	0.35
Diabetes Mellitus (%)			0.55
No	70.98 (69.16, 72.80)	70.11 (68.47, 71.75)	
Diabetes	19.91 (18.40, 21.42)	21.09 (19.58, 22.59)	
IFG	6.22 (5.12, 7.31)	5.68 (4.74, 6.62)	
IGT	2.90 (2.35, 3.44)	3.12 (2.51, 3.73)	
Hyperlipidemia (%)	77.44 (75.59, 79.30)	78.20 (76.33, 80.07)	0.59

Abbreviations: CHF, congestive heart failure; CHD, coronary heart disease; CVD, cardiovascular disease; IFG, impaired fasting glucose; IGT, impaired glucose tolerance.

^a^ Two-sided *p* values show results of univariate comparisons between participants with cancer and participants without cancer. The two-samples Student’s t*-*test was used for normally distributed variables, while Mann-Whitney U test was used for non-parametric variables. Design-based χ^2^ tests were employed to assess the associations of categorical variables with cancer status. Continuous variables with normality were presented as weighted means with associated standard errors, and variables without normality were presented as weighted median with associated interquartile range. Categorical variables were expressed as weighted proportions and corresponding 95% confidence intervals.

^b^ Categorized into the following three levels based on the poverty income ratio: low income (<1.3), middle income (1.3–3.5), and high income (≥3.5).

^c^ Categorized into the following 3 levels: never, smoked less than 100 cigarettes in life; former, smoked more than 100 cigarettes in life and smoke not at all; current, smoked more than 100 cigarettes in life and smoke some days or every day.

^d^ Divided into three categories: low (<18.5 kg/m^2^), normal (18.5–25 kg/m^2^), overweight (≥25 kg/m^2^).

### Survival Analysis

The leading causes of death for those with and without cancer are presented in Supplementary Figure S1. The weighted prevalence of all-cause, cardiovascular-related, or malignant neoplasms mortality was 26.05%, 6.48% and 8.79%, respectively, in participants with cancer, and was 9.12%, 2.79% and 1.96%, respectively, for participants without cancer (details in Supplementary Table S4).

### The Association Between Mortality Risk and Cancer and Cancer Type

The weighted Cox proportional hazards regression analysis results (Table 3 and Supplementary Table S5) for the association between cancer and the risk of cardiovascular-related and all-cause mortality consistently revealed that cancer contributes considerably to the risks of cardiovascular-related and all-cause mortality. For example, Model 3 indicated that participants with cancer had a 54% higher risk of cardiovascular-related mortality (HR, 1.54; 95% CI, 1.34–1.78; *p* < 0.0001) and 100% higher risk of all-cause mortality (HR, 2.00; 95% CI, 1.84–2.17; *p* < 0.0001), compared to participants without cancer. Matched population analysis showed that participants with cancer had a 43% higher risk of cardiovascular-related mortality (HR, 1.43; 95% CI, 1.20–1.69; *p* < 0.0001) and 92% higher risk of all-cause mortality (HR, 1.92; 95% CI, 1.74–2.13; *p* < 0.0001), compared to participants without cancer in Model 3.

**TABLE 3:** Association between cancer status with cardiovascular-related mortality (The United States, 1999–2018).

Model	Non-cancer	Cancer (before PSM)	Cancer (after PSM)
Model 1	1.00 (Reference)	3.01 (2.62, 3.46)	1.33 (1.12, 1.59)
*p* Values		<0.0001	0.001
Model 2	1.00 (Reference)	1.58 (1.37, 1.82)	1.43 (1.21, 1.69)
*p* Values		<0.0001	<0.0001
Model 3	1.00 (Reference)	1.54 (1.34, 1.78)	1.43 (1.20, 1.69)
*p* Values		<0.0001	<0.0001

Values are HR (95% CI).

Model 1: Unadjusted model; Model 2: Adjusted for age (<50 years or ≥50 years), sex (Male, Female), ethnicity (White, Non-white), marital status (Living with a spouse/partner, Living without a spouse/partner), poverty income ratio [classified as low income (<1.3), middle income (1.3–3.5), and high income (≥3.5)], educational level (divided into less than 9th grade, 9–11th grade, high school graduate, some college or AA degree, college graduate or above).

Model 3: Further adjusted for the American Heart Association’s Life’s Simple 7 cardiovascular health score (continuous).

Abbreviations: CI, confidence interval; HR, hazard ratio; PSM, propensity score matching.

**FIGURE 2:**
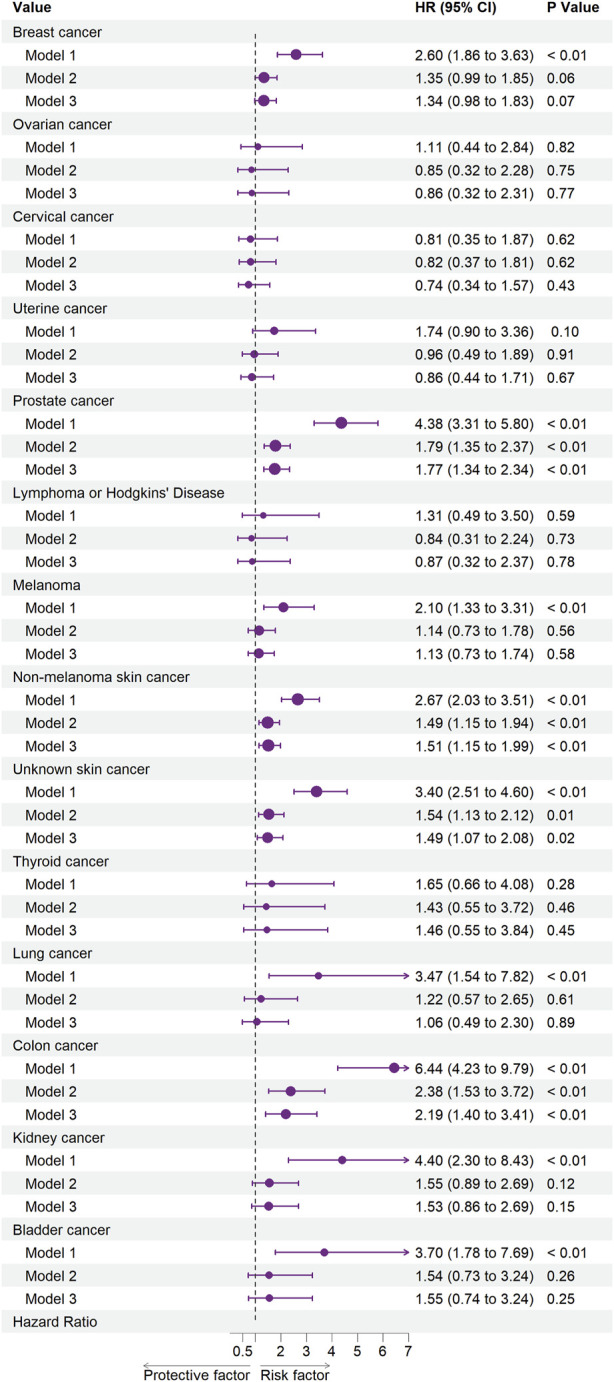
Associations between specific cancers and cardiovascular mortality. Abbreviations: CI, confidence interval; HR, hazard ratio. Each cancer type group was adjusted for age (<50 years or >50 years), sex (Male, Female), ethnicity (White, Non-white), marital status (living with a spouse/partner, or living without a spouse/partner), poverty income ratio [classified as low income (<1.3), middle income (1.3–3.5), and high income (≥3.5)], educational level (divided into less than 9th grade, 9–11th grade, high school graduate, some college or AA degree, college graduate or above) and the American Heart Association’s Life’s Simple 7 cardiovascular health score (continuous), except the stratification factor itself. Squares indicate HRs, with horizontal lines indicating 95% CIs (The United States, 1999–2018).

**FIGURE 3:**
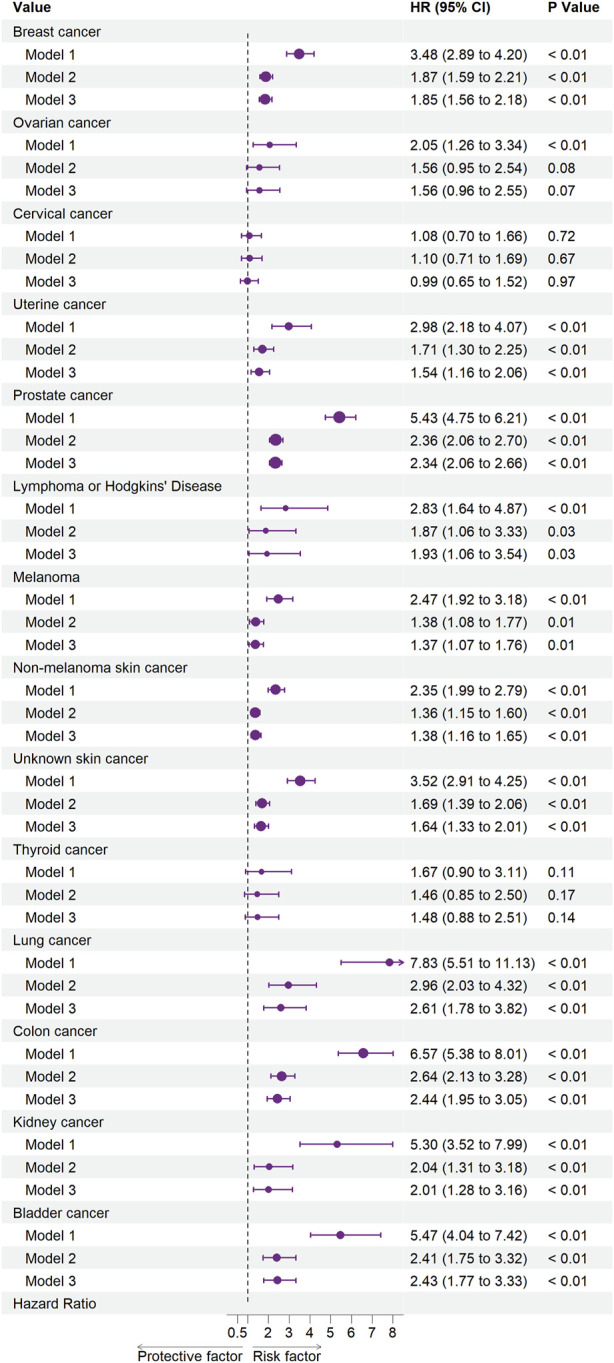
Associations between specific cancers and all-cause mortality. Abbreviations: CI, confidence interval; HR, hazard ratio. Each cancer type group was adjusted for age (<50 years or >50 years), sex (Male, Female), ethnicity (White, Non-white), marital status (living with a spouse/partner, or living without a spouse/partner), poverty income ratio [classified as low income (<1.3), middle income (1.3–3.5), and high income (≥3.5)], educational level (divided into less than 9th grade, 9–11th grade, high school graduate, some college or AA degree, college graduate or above) and the American Heart Association’s Life’s Simple 7 cardiovascular health score (continuous), except the stratification factor itself. Squares indicate HRs, with horizontal lines indicating 95% CIs (The United States, 1999–2018).

Figures 2, 3 illustrate the associations between cancer types and the risk of cardiovascular-related and all-cause mortality using the weighted Cox proportional hazards regression analysis. The fully adjusted Model 3 in Figure 2 showed that prostate cancer (HR, 1.77; 95% CI, 1.34–2.34; *p* < 0.01), non-melanoma skin cancer (HR, 1.51; 95% CI, 1.15–1.99; *p* < 0.01), unknown skin cancer (HR, 1.49; 95% CI, 1.07–2.08; *p* = 0.02), and colon cancer (HR, 2.19; 95% CI, 1.40–3.41; *p* < 0.01) increased the risk of cardiovascular-related mortality. Model 3 in Figure 3 also indicated that breast cancer (HR, 1.85; 95% CI, 1.56–2.18; *p* < 0.01), uterine cancer (HR, 1.54; 95% CI, 1.16–2.06; *p* < 0.01), prostate cancer (HR, 2.34; 95% CI, 2.06–2.66; *p* < 0.01), lymphoma or Hodgkin’s disease (HR, 1.93; 95% CI, 1.06–3.54; *p* = 0.03), melanoma (HR, 1.37; 95% CI, 1.07–1.76; *p* = 0.01), non-melanoma skin cancer (HR, 1.38; 95% CI, 1.16–1.65; *p* < 0.01), unknown skin cancer (HR, 1.64; 95% CI, 1.33–2.01; *p* < 0.01), lung cancer (HR, 2.61; 95% CI, 1.78–3.82; *p* < 0.01), colon cancer (HR, 2.44; 95% CI, 1.95–3.05; *p* < 0.01), kidney cancer (HR, 2.01; 95% CI, 1.28–3.16; *p* < 0.01), and bladder cancer (HR, 2.43; 95% CI, 1.77–3.33; *p* < 0.01) increased the risk of all-cause mortality.

### Subgroup Analyses

Supplementary Figures S2, S3 summarize the subgroup analysis results using multivariable adjusted weighted Cox proportional hazards regressions.

Supplementary Figure S2 indicates that an elevated cardiovascular-related mortality risk was associated with cancer status among older adults (aged 50–85; HR, 1.54; 95% CI, 1.34–1.78; *p* < 0.001), White (HR, 1.57; 95% CI, 1.35–1.82, *p* < 0.001), and participants whose education level was college graduate or above (HR, 1.77; 95% CI, 1.31–2.40; *p* < 0.001), some college or AA degree (HR, 1.76; 95% CI, 1.32–2.35; *p* < 0.001), or high school graduate (HR, 1.43; 95% CI, 1.08–1.91; *p* = 0.014). Moreover, subgroup analysis identified a significant interaction between cancer status and sex (P for interaction = 0.018) in relation to the risk of cardiovascular-related mortality.

Supplementary Figure S3 suggests that the all-cause mortality risk was elevated in every subgroup. Furthermore, a significant interaction was identified between cancer status and sex (P for interaction < 0.001), ethnicity (P for interaction = 0.003), and the history of hyperlipidemia (P for interaction = 0.003) in the risk of all-cause mortality.

## Discussion

This prospective analysis of a nationally representative cohort of the United States population found strong independent associations between adult cancer survivorship and the risk of all-cause and cardiovascular-related mortality. After accounting for risk factors shared by cancer and CVD, cancer survivors had an elevated risk of all-cause and cardiovascular-related mortality than those without cancer both before and after PSM. Importantly, the associations between cancer survivorship and CVD remained largely unchanged between minimally-adjusted analyses (Model 2) accounting only for the participants’ demographics and a robustly-adjusted model (Model 3) that also accounted for traditional CVD risk factors, suggesting that cancer-specific mechanisms likely contribute to the excess burden and risk of CVD in this population.

Notably, we did not exclude participants with CVD at baseline mainly based on the following considerations, because we found in previous studies and literature [2] reports that there is also an association between cancer and CVD. Moreover, it is noteworthy that this study primarily used the AHA’s Life’s Simple 7 cardiovascular health score to adjust for shared risk factors. This adjustment serves to partially account for the baseline cardiovascular risk factors present in this study, as extensive epidemiological research has demonstrated that populations and individuals who exhibit optimal cardiovascular disease risk factors and health behaviors are associated with significantly reduced rates of CVD events. It has been suggested that the elimination of risk factors among both young individuals and adults could potentially eradicate a substantial portion, potentially exceeding 70%, of the CVD epidemic in the United States. Yang et al. studied the NHANES population and found that a higher Life’s Simple 7 score was associated with a lower all-cause and CVD mortality [34]. Gaye et al. analyzed 9,294 individuals aged ≥65 years, reporting that higher AHA’s Life’s Simple 7 cardiovascular health scores were associated with lower mortality and the incidence of vascular events in this population [35]. Moreover, an analysis of patients with a history of cancer by Kaneko et al. demonstrated that Life’s Simple 7 cardiovascular health metrics could be used for risk stratification of future CVD events in cancer survivors [29]. Moreover, the 1 year change in the Life’s Simple 7 score was associated with the risk of subsequent CVD events; furthermore, that study confirmed the clinical significance of pursuing modifiable risk factors in CVD development among cancer survivors and suggested the potential clinical benefit of optimizing the Life’s Simple 7 to prevent CVD in this patient group. Therefore, Life’s Simple 7 cardiovascular health metrics is a simple and informative assessment tool for risk factors shared by cancer and CVD [36]. The associations between cancer and the risk of all-cause and cardiovascular-related mortality in this study were adjusted for the shared risk factors, so they were largely independent of the traditional CVD risk factors.

Variation in all-cause mortality risk across primary cancers suggests that the malignancy and cancer therapies were likely central to all-cause mortality risk in this population [37]. For example, breast, hematopoietic, and lymphatic cancers are typically managed with combination chemotherapy, often anthracycline-based, and chest radiation, both with well-established cardiotoxic potential [38]. Conversely, thyroid cancer may be managed with active surveillance or local therapies without a cardiotoxic risk [39]. While our study indicated a strong independent association of adult cancer survivorship with cardiovascular-related mortality risk, it did not find statistically significant associations between the risk of cardiovascular-related mortality and most cancer types, possibly due to the few cardiovascular-related mortality events and small sample size in each cancer type and the resulting lack of statistical power to detect such associations.

Apart from the insufficient statistical power, this discrepancy could result from the lack of detailed information about the systemic therapy type or radiation dosages given. Chemotherapeutic agents are not equally cardiotoxic, and radiation cardiotoxicity is dosage-dependent [40, 41]. Therefore, it is possible that grouping all chemotherapeutic agents and radiation treatments, irrespective of the type and dosage used, will result in an underestimation of their true effect. Furthermore, previous studies have shown that the risk of CVD events is cumulative over time, with most occurring many years after the cancer diagnosis [42, 43]. Hence, with the increase in follow-up duration, significant differences in the associations between cardiovascular-related mortality risk and most cancer types would be detected. Additional studies are needed to elucidate the contribution of cancer therapies to cardiovascular-related and all-cause mortality in cancer survivors.

Nonetheless, our findings have important clinical and public health implications. CVD screening and prevention practices among cancer survivors are highly variable and often neglected due to limited evidence guiding practice and misconceptions regarding competing cancer mortality risks. 8.43% (95% CI, 7.90%–8.97%) of the participants with cancer in this study were comorbid with CVD, and 3.14% (95% CI, 2.86%–3.41%) of those with cancer died of a cardiovascular-related cause, indicating that this population would likely benefit from aggressive screening and preventive interventions. However, we also demonstrated that the links between cancer and all-cause and cardiovascular-related death go beyond traditional risk factors. Therefore, while attention to risk factors shared by cancer and CVD is needed, our data suggest that traditional risk assessment tools will likely underestimate the risks in this population, and risk factor modification alone would likely be insufficient to fully address the all-cause and cardiovascular-related mortality risks in this population. Furthermore, it is important to consider the variable associations between specific cancer types and all-cause and cardiovascular-related mortality, as some adult cancer survivor subsets have a particularly high risk. Further studies are needed to inform screening and preventive strategies specific to this unique patient population.

The present study had some limitations. First, survivors with a better prognosis were more likely to be enrolled in the NHANES, given that they were recipients of a routine health examination. Therefore, the generalization of our findings to cancer survivors with poor prognoses should be made with caution. Second, applying a competitive risk model in the survival analysis could not be performed due to the complex, stratified, multistage probability cluster design of the NHANES. Third, cancer assessment based on self‐reports instead of through medical record validation might have biased the study findings. Fourth, the observational nature of the study means that we cannot eliminate the possibility of residual confounders and the cohort design of study might limit conferring causal inference between cancer and cardiovascular mortality. Fifth, even with 44,342 adults at baseline, our statistical power to detect small to moderate associations, especially in specific cancer types and demographic subgroups, was likely limited. Sixth, many records with missing data have been deleted and we did not rule out participants with baseline CVD in this cohort study, Thus, the interpretation of the final results should be with caution. Seventh, this study aimed to assess CVD mortality and all-cause mortality outcomes rather than CVD incidence. Florido et al., using CVD incidence as a study outcome, have reported that adult cancer survivors have significantly higher risk of CVD, contributing to the evidence on the primordial and primary prevention of CVD [44]. However, our study provides further evidence on the tertiary prevention of CVD. Successful tertiary prevention may provide significant long-term benefits for adult cancer survivors. Thus, our findings also have significance for etiological research. Finally, we had limited information on cancer staging, which could influence cancer treatments, and for which we did not have sufficient statistical power to perform stratified analysis. Similarly, we could not verify the effect of cancer treatment on cardiovascular-related and all-cause mortality for the lack of information on cancer treatment modalities, which might directly contribute to the observed variability in cardiovascular-related and all-cause mortality risk across cancer types.

### Conclusion

Our study confirmed that cancer was strongly linked to cardiovascular-related and all-cause mortality, even after adjusting for various factors that could impact risk, including the AHA’s Life’s Simple 7 cardiovascular health score, age, sex, ethnicity, marital status, income, and education level. Therefore, the excess burden of all-cause and cardiovascular-related mortality in this population was not fully explained by traditional cardiovascular risk factors and might be related to late effects of cancer and the cardiotoxic effects of its treatments.

## Data Availability

NHANES and the NDI linkage data are publicly available at https://www.cdc.gov/nchs/nhanes/index.htm and https://www.cdc.gov/nchs/data-linkage/mortality-public.htm. We intend to provide relevant code on written reasonable request. Dissemination to study participants and/or patient organizations is not possible/applicable given the nature of public use and deidentified NHANES and NDI data.

## Supplementary Material

Supplementary Figure S1 The weighted prevalence of leading causes of death in study participants according to cancer status.

Supplementary Figure S2 Association between cancer status and cardiovascular mortality, overall and by subgroups of interest. Abbreviations: CI, confidence interval; HR, hazard ratio. Each stratification was adjusted for age (<50 years or >50 years), sex (Male, Female), ethnicity (White, Non-white), marital status (living with a spouse/partner, or living without a spouse/partner), poverty income ratio [classified as low income (<1.3), middle income (1.3–3.5), and high income (≥3.5)], educational level (divided into less than 9th grade, 9–11th grade, high school graduate, some college or AA degree, college graduate or above) and the American Heart Association’s Life’s Simple 7 cardiovascular health score (continuous), except the stratification factor itself. Squares indicate HRs, with horizontal lines indicating 95% CIs. Diamonds indicate overall HRs, with outer points of the diamonds indicating 95% CIs.

Supplementary Figure S3 Association between cancer status and all-cause mortality, overall and by subgroups of interest. Abbreviations: CI, confidence interval; HR, hazard ratio. Each stratification was adjusted for age (<50 years or > 50 years), sex (Male, Female), ethnicity (White, Non-white), marital status (living with a spouse/partner, or living without a spouse/partner), poverty income ratio (classified as low income (<1.3), middle income (1.3–3.5), and high income (≥3.5)], educational level (divided into less than 9th grade, 9–11th grade, high school graduate, some college or AA degree, college graduate or above) and the American Heart Association’ s Life’ s Simple 7 cardiovascular health score (continuous), except the stratification factor itself. Squares indicate HRs, with horizontal lines indicating 95% CIs. Diamonds indicate overall HRs, with outer points of the diamonds indicating 95% CIs.
