# Supplementary figures and images for: The Role of Cancer in the Risk of Cardiovascular and All-Cause Mortality: A Nationwide Prospective Cohort Study
Source: Int J Public Health. 2023 Oct 19;68:1606088. doi: 10.3389/ijph.2023.1606088 (PMC10620309; doi:10.3389/ijph.2023.1606088)

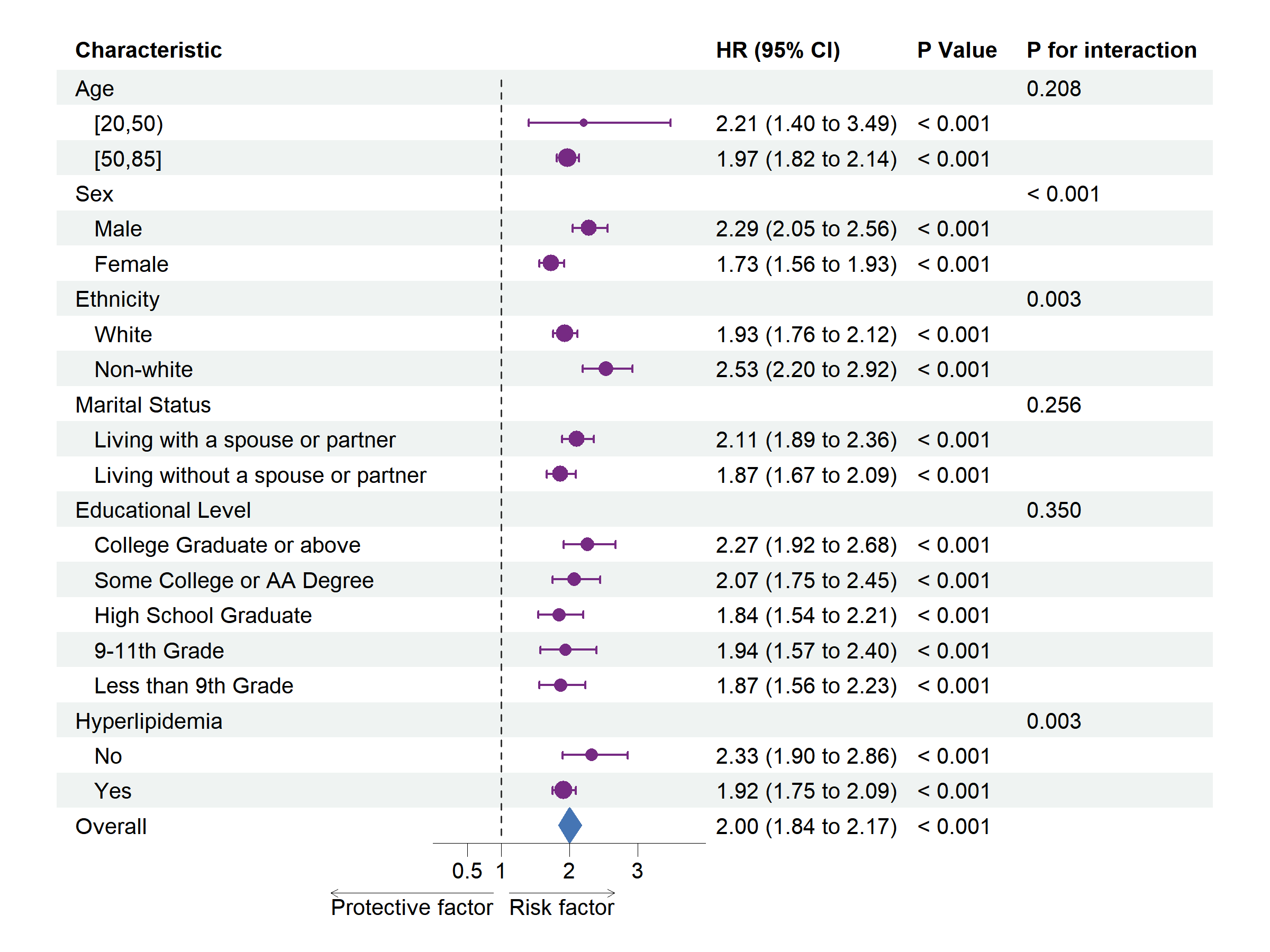

Supplement: Supplementary file 1 [file Image3.TIFF]

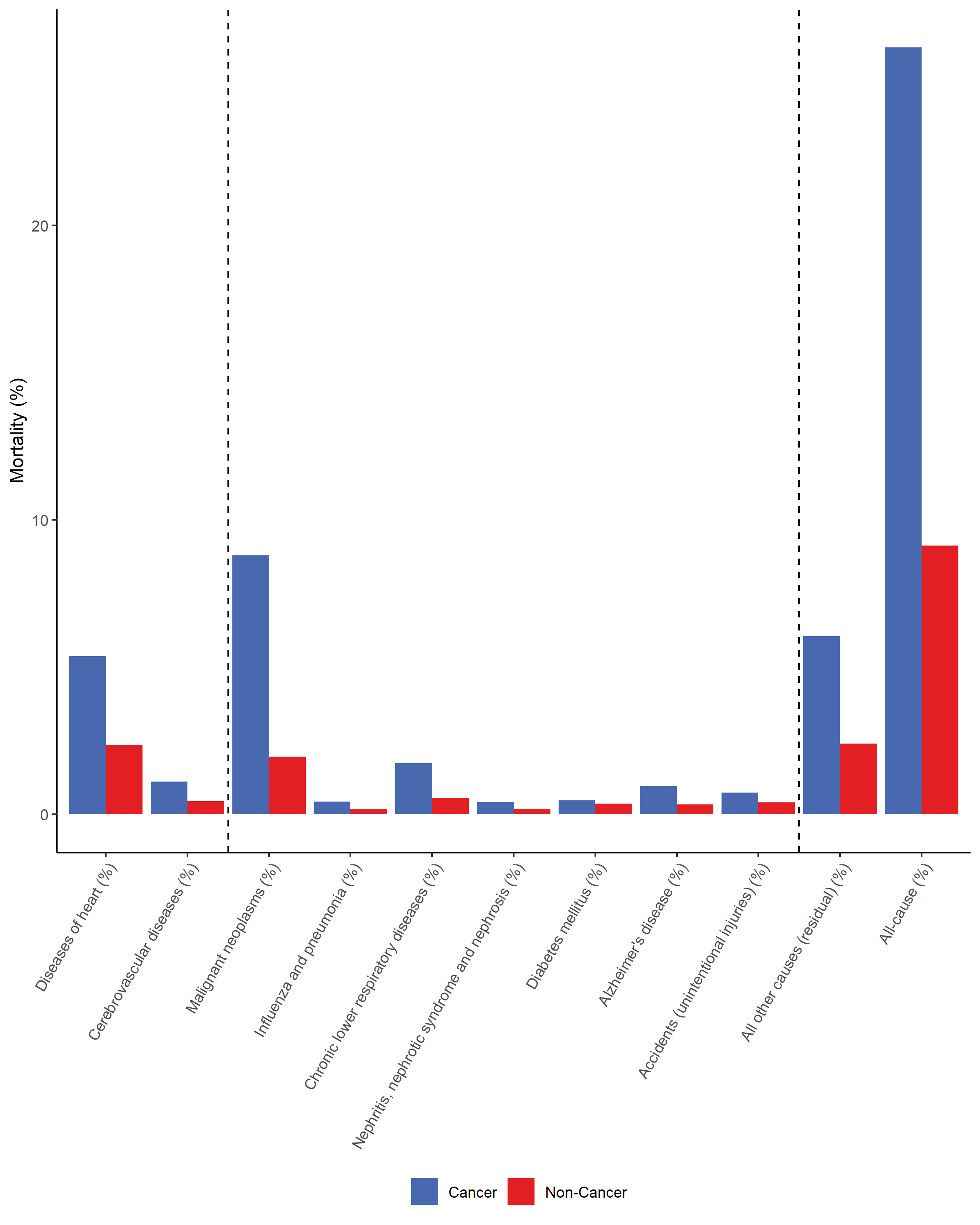

Supplement: Supplementary file 2 [file Image1.TIFF]

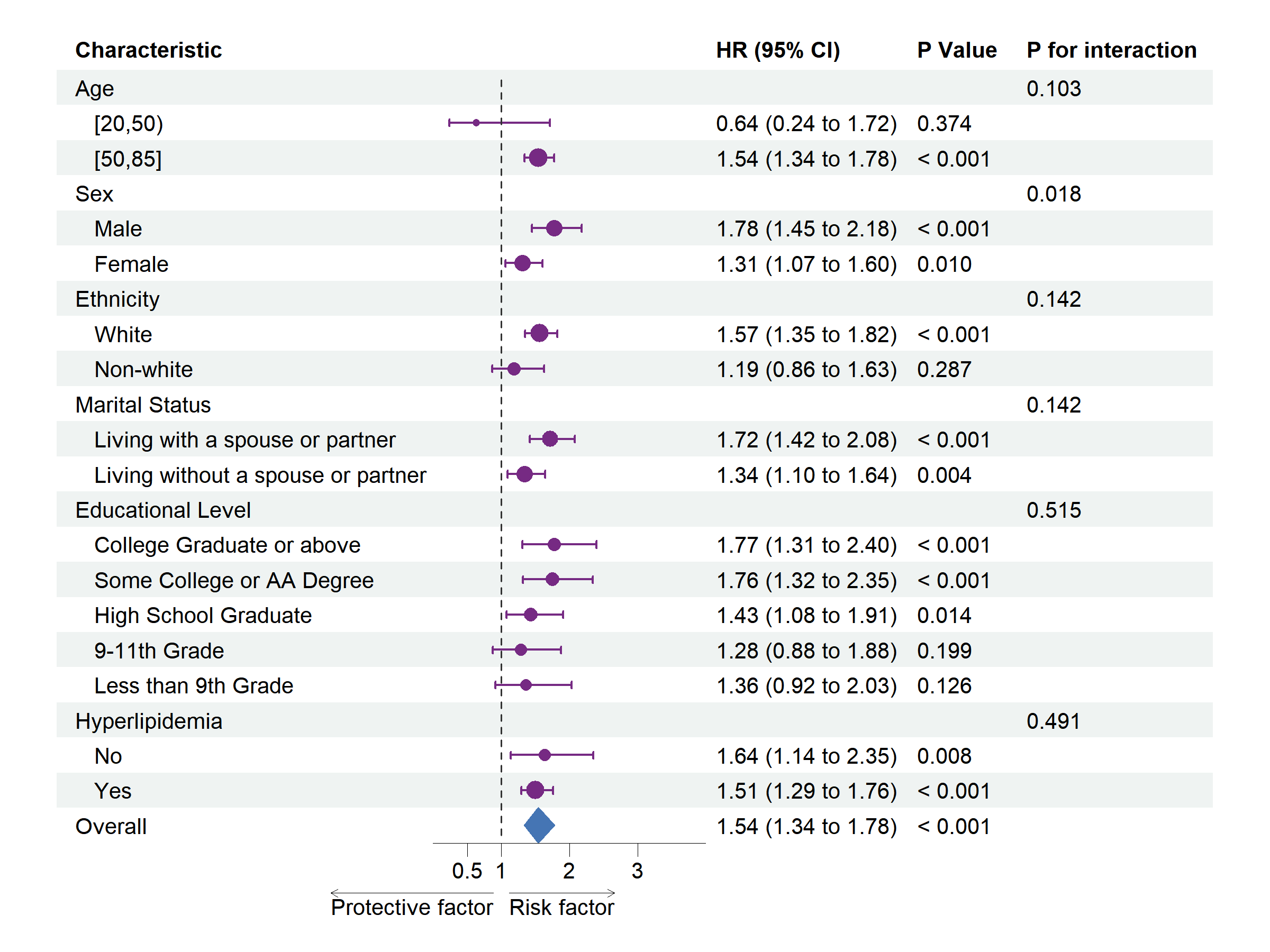

Supplement: Supplementary file 3 [file Image2.TIFF]
