# Supplementary material for: The Role of Cancer in the Risk of Cardiovascular and All-Cause Mortality: A Nationwide Prospective Cohort Study
Source: Int J Public Health. 2023 Oct 19;68:1606088. doi: 10.3389/ijph.2023.1606088 (PMC10620309; doi:10.3389/ijph.2023.1606088)
Supplement: Supplementary file 4 [file DataSheet1.docx]

**Supplemental Table 1.** Summary of Balance for All Data Prior to Propensity Score Matching

| Covariates | Means Treated | Means Control | SMD | variance ratios | eCDF Mean | eCDF Max |
| --- | --- | --- | --- | --- | --- | --- |
| Distance | 0.2024 | 0.0837 | 0.9308 | 1.9271 | 0.2850 | 0.4637 |
| Age | 62.1737 | 45.2943 | 1.1618 | 0.8104 | 0.2557 | 0.4297 |
| Sex |  |  |  |  |  |  |
| Female | 0.5705 | 0.5062 | 0.1300 | **-** | 0.0643 | 0.0643 |
| Male | 0.4295 | 0.4938 | -0.1300 | **-** | 0.0643 | 0.0643 |
| Ethnicity |  |  |  |  |  |  |
| White | 0.8701 | 0.6741 | 0.5830 | **-** | 0.1960 | 0.1960 |
| Non-white | 0.1299 | 0.3259 | -0.5830 | **-** | 0.1960 | 0.1960 |
| Marital Status |  |  |  |  |  |  |
| Living with a Spouse or Partner | 0.6650 | 0.6362 | 0.0609 | **-** | 0.0288 | 0.0288 |
| Living without a Spouse or Partner | 0.3350 | 0.3638 | -0.0609 | **-** | 0.0288 | 0.0288 |
| Poverty income ratio | 3.2463 | 2.9795 | 0.1683 | 0.9322 | 0.0532 | 0.0708 |
| Educational Level |  |  |  |  |  |  |
| College Graduate or above | 0.3228 | 0.2785 | 0.0947 | **-** | 0.0443 | 0.0443 |
| Some College or AA Degree | 0.3122 | 0.3110 | 0.0026 | **-** | 0.0012 | 0.0012 |
| High School Graduate | 0.2237 | 0.2418 | -0.0435 | **-** | 0.0181 | 0.0181 |
| 9-11th Grade | 0.0923 | 0.1122 | -0.0688 | **-** | 0.0199 | 0.0199 |
| Less than 9th Grade | 0.0490 | 0.0565 | -0.0346 | **-** | 0.0075 | 0.0075 |
| Smoking Status |  |  |  |  |  |  |
| Never | 0.4427 | 0.5465 | -0.2091 | **-** | 0.1038 | 0.1038 |
| Former | 0.3896 | 0.2320 | 0.3233 | **-** | 0.1577 | 0.1577 |
| Current | 0.1677 | 0.2215 | -0.1441 | **-** | 0.0538 | 0.0538 |
| Body Mass Index | 28.7641 | 28.7759 | -0.0018 | 0.9157 | 0.0044 | 0.0210 |
| Hypertension |  |  |  |  |  |  |
| No | 0.4218 | 0.6535 | -0.4692 | **-** | 0.2317 | 0.2317 |
| Yes | 0.5782 | 0.3465 | 0.4692 | **-** | 0.2317 | 0.2317 |
| CVD |  |  |  |  |  |  |
| No | 0.8067 | 0.9271 | -0.3048 | **-** | 0.1204 | 0.1204 |
| Yes | 0.1933 | 0.0729 | 0.3048 | **-** | 0.1204 | 0.1204 |
| Diabetes Mellitus |  |  |  |  |  |  |
| No | 0.7011 | 0.8187 | -0.2568 | **-** | 0.1175 | 0.1175 |
| Diabetes | 0.2109 | 0.1171 | 0.2300 | **-** | 0.0938 | 0.0938 |
| IFG | 0.0568 | 0.0410 | 0.0682 | **-** | 0.0158 | 0.0158 |
| IGT | 0.0312 | 0.0232 | 0.0457 | **-** | 0.0079 | 0.0079 |
| Hyperlipidemia |  |  |  |  |  |  |
| No | 0.2180 | 0.3250 | -0.2592 | **-** | 0.1070 | 0.1070 |
| Yes | 0.7820 | 0.6750 | 0.2592 | **-** | 0.1070 | 0.1070 |
| Life's Simple 7 | 7.3251 | 8.0790 | -0.3241 | 0.9252 | 0.0503 | 0.1422 |

Abbreviation: CVD, cardiovascular disease; eCDF, empirical cumulative density function; IFG, impaired fasting glucose; IGT, impaired glucose tolerance; SMD, standardized mean differences.

**Supplemental Table 2.** Summary of Balance for Data after Propensity Score Matching

| Covariates | Means Treated | Means Control | SMD | variance ratios | eCDF Mean | eCDF Max | standardized pair difference |
| --- | --- | --- | --- | --- | --- | --- | --- |
| Distance | 0.2024 | 0.1995 | 0.0231 | 1.0135 | 0.0042 | 0.0189 | 0.0012 |
| Age | 62.1737 | 61.8582 | 0.0217 | 1.0184 | 0.0087 | 0.0272 | 0.5046 |
| Sex |  |  |  |  |  |  |  |
| Female | 0.5705 | 0.5900 | -0.0394 | **-** | 0.0195 | 0.0195 | 1.0069 |
| Male | 0.4295 | 0.4100 | 0.0394 | **-** | 0.0195 | 0.0195 | 1.0069 |
| Ethnicity |  |  |  |  |  |  |  |
| White | 0.8701 | 0.8687 | 0.0041 | **-** | 0.0014 | 0.0014 | 0.8918 |
| Non-white | 0.1299 | 0.1313 | -0.0041 | **-** | 0.0014 | 0.0014 | 0.8918 |
| Marital Status |  |  |  |  |  |  |  |
| Living with a Spouse or Partner | 0.6650 | 0.6636 | 0.0030 | **-** | 0.0014 | 0.0014 | 0.9840 |
| Living without a Spouse or Partner | 0.3350 | 0.3364 | -0.0030 | **-** | 0.0014 | 0.0014 | 0.9840 |
| Poverty income ratio | 3.2463 | 3.2259 | 0.0128 | 1.0133 | 0.0065 | 0.0204 | 1.1314 |
| Educational Level |  |  |  |  |  |  |  |
| College Graduate or above | 0.3228 | 0.3094 | 0.0288 | **-** | 0.0134 | 0.0134 | 0.8083 |
| Some College or AA Degree | 0.3122 | 0.3157 | -0.0076 | **-** | 0.0035 | 0.0035 | 0.9201 |
| High School Graduate | 0.2237 | 0.2337 | -0.0241 | **-** | 0.0100 | 0.0100 | 0.8357 |
| 9-11th Grade | 0.0923 | 0.0972 | -0.0170 | **-** | 0.0049 | 0.0049 | 0.7862 |
| Less than 9th Grade | 0.0490 | 0.0440 | 0.0232 | **-** | 0.0050 | 0.0050 | 0.8106 |
| Smoking Status |  |  |  |  |  |  |  |
| Never | 0.4427 | 0.4393 | 0.0068 | **-** | 0.0034 | 0.0034 | 0.9467 |
| Former | 0.3896 | 0.3835 | 0.0126 | **-** | 0.0062 | 0.0062 | 0.8669 |
| Current | 0.1677 | 0.1772 | -0.0255 | **-** | 0.0095 | 0.0095 | 0.7026 |
| Body Mass Index | 28.7641 | 28.7122 | 0.0080 | 1.1217 | 0.0085 | 0.0231 | 1.0379 |
| Hypertension |  |  |  |  |  |  |  |
| No | 0.4218 | 0.4354 | -0.0275 | **-** | 0.0136 | 0.0136 | 0.8438 |
| Yes | 0.5782 | 0.5646 | 0.0275 | **-** | 0.0136 | 0.0136 | 0.8438 |
| CVD |  |  |  |  |  |  |  |
| No | 0.8067 | 0.8006 | 0.0156 | **-** | 0.0062 | 0.0062 | 0.8515 |
| Yes | 0.1933 | 0.1994 | -0.0156 | **-** | 0.0062 | 0.0062 | 0.8515 |
| Diabetes Mellitus |  |  |  |  |  |  |  |
| No | 0.7011 | 0.7098 | -0.0189 | **-** | 0.0087 | 0.0087 | 0.9662 |
| Diabetes | 0.2109 | 0.1991 | 0.0289 | **-** | 0.0118 | 0.0118 | 0.9140 |
| IFG | 0.0568 | 0.0622 | -0.0231 | **-** | 0.0053 | 0.0053 | 0.4415 |
| IGT | 0.0312 | 0.0290 | 0.0129 | **-** | 0.0022 | 0.0022 | 0.3799 |
| Hyperlipidemia |  |  |  |  |  |  |  |
| No | 0.2180 | 0.2256 | -0.0183 | **-** | 0.0076 | 0.0076 | 0.8587 |
| Yes | 0.7820 | 0.7744 | 0.0183 | **-** | 0.0076 | 0.0076 | 0.8587 |
| Life's Simple 7 | 7.3251 | 7.3722 | -0.0203 | 1.0026 | 0.0037 | 0.0159 | 1.0768 |

Abbreviation: CVD, cardiovascular disease; eCDF, empirical cumulative density function; IFG, impaired fasting glucose; IGT, impaired glucose tolerance; SMD, standardized mean differences.

**Supplemental Table 3.** Percent Balance Improvement (%)

| Covariates | SMD | variance ratios | eCDF Mean | eCDF Max |
| --- | --- | --- | --- | --- |
| Distance | 97.5 | 97.9 | 98.5 | 95.9 |
| Age | 98.1 | 91.3 | 96.6 | 93.7 |
| Sex |  |  |  |  |
| Female | 69.7 | **-** | 69.7 | 69.7 |
| Male | 69.7 | **-** | 69.7 | 69.7 |
| Ethnicity |  |  |  |  |
| White | 99.3 | **-** | 99.3 | 99.3 |
| Non-white | 99.3 | **-** | 99.3 | 99.3 |
| Marital Status |  |  |  |  |
| Living with a Spouse or Partner | 95.1 | **-** | 95.1 | 95.1 |
| Living without a Spouse or Partner | 95.1 | **-** | 95.1 | 95.1 |
| Poverty income ratio | 92.4 | 81.2 | 87.8 | 71.1 |
| Educational Level |  |  |  |  |
| College Graduate or above | 69.6 | **-** | 69.6 | 69.6 |
| Some College or AA Degree | -185.9 | **-** | -185.9 | -185.9 |
| High School Graduate | 44.6 | **-** | 44.6 | 44.6 |
| 9-11th Grade | 75.3 | **-** | 75.3 | 75.3 |
| Less than 9th Grade | 32.9 | **-** | 32.9 | 32.9 |
| Smoking Status |  |  |  |  |
| Never | 96.7 | **-** | 96.7 | 96.7 |
| Former | 96.1 | **-** | 96.1 | 96.1 |
| Current | 82.3 | **-** | 82.3 | 82.3 |
| Body Mass Index | -341.1 | -30.5 | -90.5 | -10.1 |
| Hypertension |  |  |  |  |
| No | 94.1 | **-** | 94.1 | 94.1 |
| Yes | 94.1 | **-** | 94.1 | 94.1 |
| CVD |  |  |  |  |
| No | 94.9 | **-** | 94.9 | 94.9 |
| Yes | 94.9 | **-** | 94.9 | 94.9 |
| Diabetes Mellitus |  |  |  |  |
| No | 92.6 | **-** | 92.6 | 92.6 |
| Diabetes | 87.5 | **-** | 87.5 | 87.5 |
| IFG | 66.1 | **-** | 66.1 | 66.1 |
| IGT | 71.8 | **-** | 71.8 | 71.8 |
| Hyperlipidemia |  |  |  |  |
| No | 92.9 | **-** | 92.9 | 92.9 |
| Yes | 92.9 | **-** | 92.9 | 92.9 |
| Life's Simple 7 | 93.8 | 96.6 | 92.6 | 88.8 |

Abbreviation: CVD, cardiovascular disease; eCDF, empirical cumulative density function; IFG, impaired fasting glucose; IGT, impaired glucose tolerance; SMD, standardized mean differences.

**Supplemental Table 4.** The Weighted Prevalence of Leading Causes of Death

| Cause of death | Cancer | Non-Cancer |
| --- | --- | --- |
| Diseases of heart (I00-I09, I11, I13, I20-I51) (%) | 5.37 | 2.35 |
| Cerebrovascular diseases (I60-I69) (%) | 1.11 | 0.44 |
| Malignant neoplasms (C00-C97) (%) | 8.79 | 1.96 |
| Influenza and pneumonia (J09-J18) (%) | 0.43 | 0.16 |
| Chronic lower respiratory diseases (J40-J47) (%) | 1.73 | 0.54 |
| Nephritis, nephrotic syndrome and nephrosis (N00-N07, N17-N19, N25-N27) (%) | 0.41 | 0.18 |
| Diabetes mellitus (E10-E14) (%) | 0.47 | 0.36 |
| Alzheimer's disease (G30) (%) | 0.96 | 0.33 |
| Accidents (unintentional injuries) (V01-X59, Y85-Y86) (%) | 0.73 | 0.40 |
| All other causes (residual) (%) | 6.05 | 2.40 |
| All‑cause (%) | 26.05 | 9.12 |
| Censored (%) | 73.95 | 90.88 |

**Supplemental Table 5.** Association between Cancer Status and All-cause Mortality

| Model | Non-cancer | Cancer (before PSM) | Cancer (after PSM) |
| --- | --- | --- | --- |
| Model 1 | 1.00 (Reference) | 3.68(3.41,3.98) | 1.81(1.62,2.02) |
| P Values |  | <0.0001 | <0.0001 |
| Model 2 | 1.00 (Reference) | 2.04(1.89, 2.21) | 1.93(1.74, 2.13) |
| P Values |  | <0.0001 | <0.0001 |
| Model 3 | 1.00 (Reference) | 2.00(1.84,2.17) | 1.92(1.74, 2.13) |
| P Values |  | <0.0001 | <0.0001 |

Values are HR (95% CI).

Model 1: Unadjusted model;

Model 2: Adjusted for age (< 50years or ≥ 50years), sex (Male, Female), ethnicity (White, Non-white), marital status (Living with a spouse / partner, Living without a spouse / partner), poverty income ratio (classified as low income (<1.3), middle income (1.3-3.5), and high income (≥3.5)), educational level (divided into less than 9th grade, 9-11th grade, high school graduate, some college or AA degree, college graduate or above);

Model 3: Further adjusted for the American Heart Association’s [Life’s Simple 7](https://www.heart.org/en/healthy-living/healthy-lifestyle/my-life-check--lifes-simple-7) cardiovascular health score (continuous).

Abbreviations: CI, confidence interval; HR, hazard ratio; PSM, propensity score matching.
